# (*R*)-2-[(*R*)-2,2-Dimethyl-1,3-dioxolan-4-yl]-1,3-oxathio­lan-5-one

**DOI:** 10.1107/S160053680902039X

**Published:** 2009-06-06

**Authors:** Qin-Pei Wu, Da-Xin Shi, Hao Wang, Qing-Shan Zhang

**Affiliations:** a School of Chemical Engineering and Environment, Beijing Institute of Technology, Beijing 100081, People’s Republic of China; b School of Life Science, Beijing Institute of Technology, Beijing 100081, People’s Republic of China

## Abstract

In the title compound, C_8_H_12_O_4_S, the two five-membered rings both adopt envelope conformations. In the crystal, weak C—H⋯O inter­actions link neighbouring mol­ecules.

## Related literature

The title compound is a precursor for the preparation of an important nucleoside drug. For applications of nucleosides in the fields of biology, drugs and chemistry, see: Goodyear *et al.* (2005 ▶); Simons (2001 ▶); Vittori *et al.* (2006 ▶).
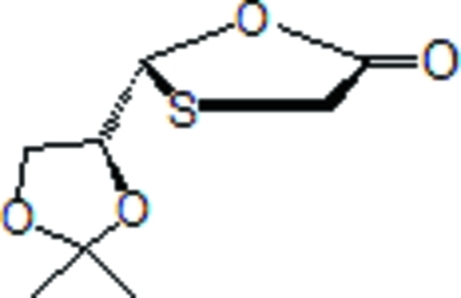


## Experimental

### 

#### Crystal data

C_8_H_12_O_4_S
*M* *_r_* = 204.24
Monoclinic, 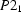

*a* = 6.5528 (13) Å
*b* = 9.4029 (19) Å
*c* = 7.9240 (16) Å
β = 106.60 (3)°
*V* = 467.89 (16) Å^3^
*Z* = 2
Mo *K*α radiation
μ = 0.33 mm^−1^
*T* = 293 K
0.50 × 0.20 × 0.15 mm

#### Data collection

Rigaku Saturn CCD area-detector diffractometer
Absorption correction: multi-scan (*CrystalClear*; Rigaku/MSC, 2005 ▶) *T*_min_ = 0.859, *T*_max_ = 0.952
1941 measured reflections
1705 independent reflections
1275 reflections with *I* > 2σ(*I*)
*R*_int_ = 0.056

#### Refinement

*R*[*F* ^2^ > 2σ(*F* ^2^)] = 0.042
*wR*(*F* ^2^) = 0.129
*S* = 1.01
1705 reflections
119 parameters
1 restraint
H-atom parameters constrained
Δρ_max_ = 0.24 e Å^−3^
Δρ_min_ = −0.20 e Å^−3^
Absolute structure: Flack (1983 ▶), 593 Friedel pairs
Flack parameter: −0.01 (13)

### 

Data collection: *RAPID-AUTO* (Rigaku/MSC, 2005 ▶); cell refinement: *RAPID-AUTO*; data reduction: *CrystalStructure* (Rigaku/MSC, 2005 ▶); program(s) used to solve structure: *SHELXS97* (Sheldrick, 2008 ▶); program(s) used to refine structure: *SHELXL97* (Sheldrick, 2008 ▶); molecular graphics: *SHELXTL* (Sheldrick, 2008 ▶); software used to prepare material for publication: *SHELXL97*.

## Supplementary Material

Crystal structure: contains datablocks global, I. DOI: 10.1107/S160053680902039X/wn2324sup1.cif

Structure factors: contains datablocks I. DOI: 10.1107/S160053680902039X/wn2324Isup2.hkl

Additional supplementary materials:  crystallographic information; 3D view; checkCIF report

## Figures and Tables

**Table 1:** Hydrogen-bond geometry (Å, °)

*D*—H⋯*A*	*D*—H	H⋯*A*	*D*⋯*A*	*D*—H⋯*A*
C1—H1*A*⋯O3^i^	0.97	2.58	3.428 (4)	146
C1—H1*B*⋯O2^ii^	0.97	2.41	3.306 (6)	153
C3—H3⋯O2^iii^	0.98	2.55	3.265 (4)	129

Symmetry codes: (i) 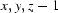; (ii) 
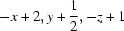
; (iii) 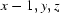.

**Fig. 1.**
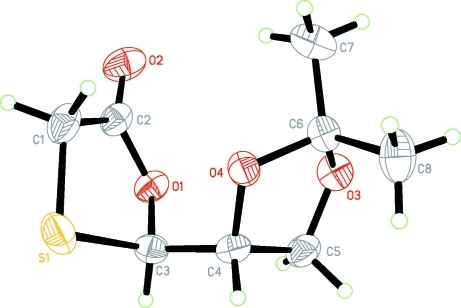
Molecular structure of the title compound with displacement ellipsoids drawn at the 30% probability level. Hydrogen atoms are drawn as spheres of arbitrary radius.

**Fig. 2.**
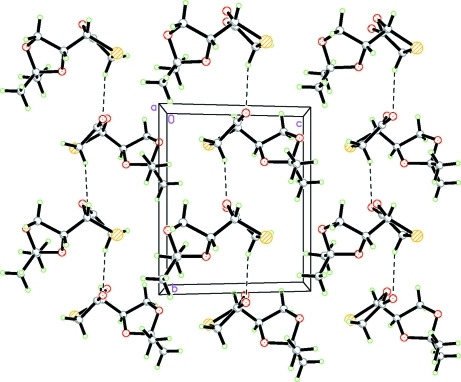
The crystal packing of the title compound, viewed along the a axis. Hydrogen bonds are shown as dashed lines.

## supplementary crystallographic information

### Comment

Nucleosides are a very important series of compounds in the fields of biology, drugs and chemistry (Simons, 2001); as an example, lamivudine is used as a drug for HIV and HBV diseases (Goodyear *et al.*, 2005; Vittori *et al.*, 2006). Studies of the synthesis of nucleoside mimetics are essential.

The purpose of this structure determination was to establish the molecular conformation of the title compound obtained by coupling (*R*)-(+)-2,2-dimethyl-1,3-dioxolane-4-carboxaldehyde with 2-mercaptoacetic acid. The chirality at the 2-position (C3) is *R*; this satisfies our requirements for the preparation of corresponding *L*-nucleosides. All bond lengths and bond angles have expected values. The two 5-membered rings both adopt envelope conformations with atoms C3 and C6 at the flap. Three intermolecular C—H···O interactions link neighbouring molecules.

### Experimental

A solution of (*R*)-(+)-2,2-dimethyl -1,3-dioxolane-4-carboxaldehyde (6.51 g, 50.0 mmol) and 2-mercaptoacetic acid (4.20 ml, 60.0 mmol) in toluene (200 ml) was heated under reflux for 1.5 h. After the reaction mixture was cooled to room temperature, a saturated aqueous solution of NaHCO_3_ (30 ml) was added and these two layers were separated. The organic layer was washed with brine, dried (MgSO_4_) and concentrated under reduced pressure. The residue was isolated through short column chromatography on silica gel, which was eluted with EtOAc-petroleum to give the target compound (4.96 g, 48%). m.p. 75–77°C.

50 mg of the final product was dissolved in petroleum ether (5 ml) and the solution was kept at room temperature for 2 days to give colorless single crystals.

### Refinement

H atoms were included in the riding model approximation, with C—H distances 0.96–0.98 Å, and with *U*_iso_(H) = k*U*_eq_(C), where k = 1.5 for methyl H and 1.2 for all other H atoms.

### Crystal data

**Table:**

C_8_H_12_O_4_S	*F*(000) = 216
*M**_r_* = 204.24	*D*_x_ = 1.450 Mg m^−^^3^
Monoclinic, *P*2_1_	Mo *K*α radiation, λ = 0.71073 Å
*a* = 6.5528 (13) Å	Cell parameters from 1941 reflections
*b* = 9.4029 (19) Å	θ = 2.7–27.5°
*c* = 7.9240 (16) Å	µ = 0.33 mm^−^^1^
β = 106.60 (3)°	*T* = 293 K
*V* = 467.89 (16) Å^3^	Block, colourless
*Z* = 2	0.50 × 0.20 × 0.15 mm

### Data collection

**Table:**

Rigaku Saturn CCD area-detector diffractometer	1705 independent reflections
Radiation source: fine-focus sealed tube	1275 reflections with *I* > 2σ(*I*)
graphite	*R*_int_ = 0.056
Detector resolution: 10.00 pixels mm^-1^	θ_max_ = 27.5°, θ_min_ = 2.7°
Ω scans	*h* = −8→8
Absorption correction: multi-scan (*CrystalClear*; Rigaku/MSC, 2005)	*k* = −12→11
*T*_min_ = 0.859, *T*_max_ = 0.952	*l* = −10→10
1941 measured reflections	

### Refinement

**Table:**

Refinement on *F*^2^	Hydrogen site location: inferred from neighbouring sites
Least-squares matrix: full	H-atom parameters constrained
*R*[*F*^2^ > 2σ(*F*^2^)] = 0.042	*w* = 1/[σ^2^(*F*_o_^2^) + (0.088*P*)^2^] where *P* = (*F*_o_^2^ + 2*F*_c_^2^)/3
*wR*(*F*^2^) = 0.129	(Δ/σ)_max_ < 0.001
*S* = 1.01	Δρ_max_ = 0.24 e Å^−^^3^
1705 reflections	Δρ_min_ = −0.20 e Å^−^^3^
119 parameters	Extinction correction: *SHELXL97* (Sheldrick, 2008), Fc^*^=kFc[1+0.001xFc^2^λ^3^/sin(2θ)]^-1/4^
1 restraint	Extinction coefficient: 0.102 (15)
Primary atom site location: structure-invariant direct methods	Absolute structure: Flack (1983), 593 Friedel pairs
Secondary atom site location: difference Fourier map	Flack parameter: −0.01 (13)

### Special details

**Table:**

Experimental. ^1^H NMR(CDCl_3_,*P*.P.*M*.): 1.41 (d, 6 H), 3.58(d, 4 H), 3.77 (d, 1 H), 3.92 (dd, 1 H), 4.12 (dd, 1 H), 4.35 (m, 1 H), 5.45(d, 1 H).
Geometry. All e.s.d.'s (except the e.s.d. in the dihedral angle between two l.s. planes) are estimated using the full covariance matrix. The cell e.s.d.'s are taken into account individually in the estimation of e.s.d.'s in distances, angles and torsion angles; correlations between e.s.d.'s in cell parameters are only used when they are defined by crystal symmetry. An approximate (isotropic) treatment of cell e.s.d.'s is used for estimating e.s.d.'s involving l.s. planes.
Refinement. Refinement of *F*^2^ against ALL reflections. The weighted *R*-factor *wR* and goodness of fit *S* are based on *F*^2^, conventional *R*-factors *R* are based on *F*, with *F* set to zero for negative *F*^2^. The threshold expression of *F*^2^ > σ(*F*^2^) is used only for calculating *R*-factors(gt) *etc*. and is not relevant to the choice of reflections for refinement. *R*-factors based on *F*^2^ are statistically about twice as large as those based on *F*, and *R*- factors based on ALL data will be even larger.

### Fractional atomic coordinates and isotropic or equivalent isotropic displacement parameters (Å^2^)

**Table:**

	*x*	*y*	*z*	*U*_iso_*/*U*_eq_	
S1	0.57296 (17)	0.19608 (12)	0.31212 (10)	0.0733 (4)	
O1	0.7766 (4)	0.0255 (3)	0.5605 (3)	0.0514 (6)	
O2	1.1103 (4)	0.0457 (3)	0.5630 (4)	0.0734 (8)	
O3	0.8026 (4)	0.1607 (2)	0.9331 (3)	0.0560 (6)	
O4	0.7352 (4)	0.2936 (2)	0.6920 (3)	0.0482 (5)	
C1	0.8546 (6)	0.1980 (5)	0.3737 (4)	0.0612 (9)	
H1A	0.9049	0.1803	0.2718	0.073*	
H1B	0.9075	0.2899	0.4225	0.073*	
C2	0.9307 (6)	0.0859 (4)	0.5061 (4)	0.0502 (8)	
C3	0.5800 (5)	0.1011 (4)	0.5101 (4)	0.0509 (8)	
H3	0.4622	0.0328	0.4851	0.061*	
C4	0.5669 (5)	0.1947 (4)	0.6584 (4)	0.0486 (7)	
H4	0.4302	0.2451	0.6269	0.058*	
C5	0.5987 (5)	0.1192 (5)	0.8312 (4)	0.0567 (9)	
H5A	0.4914	0.1477	0.8871	0.068*	
H5B	0.5915	0.0169	0.8144	0.068*	
C6	0.8418 (5)	0.2954 (4)	0.8756 (4)	0.0486 (8)	
C7	1.0731 (6)	0.3117 (5)	0.9004 (6)	0.0742 (11)	
H7A	1.1010	0.4044	0.8614	0.111*	
H7B	1.1201	0.2405	0.8330	0.111*	
H7C	1.1483	0.3006	1.0228	0.111*	
C8	0.7487 (7)	0.4089 (5)	0.9608 (5)	0.0743 (12)	
H8A	0.7778	0.5000	0.9182	0.111*	
H8B	0.8106	0.4046	1.0861	0.111*	
H8C	0.5975	0.3954	0.9332	0.111*	

### Atomic displacement parameters (Å^2^)

**Table:**

	*U*^11^	*U*^22^	*U*^33^	*U*^12^	*U*^13^	*U*^23^
S1	0.0745 (6)	0.0908 (9)	0.0459 (4)	−0.0108 (6)	0.0034 (4)	0.0026 (5)
O1	0.0541 (13)	0.0404 (12)	0.0659 (14)	−0.0020 (10)	0.0269 (11)	−0.0011 (11)
O2	0.0606 (17)	0.0628 (18)	0.107 (2)	0.0052 (14)	0.0402 (16)	−0.0045 (16)
O3	0.0649 (15)	0.0446 (14)	0.0563 (12)	0.0047 (11)	0.0139 (10)	0.0108 (11)
O4	0.0566 (12)	0.0465 (12)	0.0411 (10)	−0.0113 (11)	0.0132 (9)	0.0000 (9)
C1	0.082 (2)	0.053 (2)	0.0589 (18)	−0.012 (2)	0.0367 (17)	−0.0009 (17)
C2	0.059 (2)	0.0397 (17)	0.0597 (18)	−0.0016 (15)	0.0293 (16)	−0.0084 (14)
C3	0.0457 (16)	0.0508 (19)	0.0557 (17)	−0.0117 (15)	0.0134 (14)	−0.0050 (16)
C4	0.0389 (14)	0.0522 (19)	0.0563 (15)	−0.0025 (15)	0.0163 (12)	0.0032 (17)
C5	0.0558 (19)	0.060 (2)	0.062 (2)	−0.0076 (17)	0.0287 (17)	0.0027 (16)
C6	0.0548 (19)	0.0429 (18)	0.0453 (16)	0.0041 (15)	0.0100 (14)	0.0022 (13)
C7	0.058 (2)	0.065 (3)	0.088 (3)	−0.0082 (19)	0.004 (2)	−0.004 (2)
C8	0.106 (3)	0.057 (2)	0.063 (2)	0.015 (2)	0.027 (2)	−0.0093 (18)

### Geometric parameters (Å, °)

**Table:**

S1—C1	1.769 (4)	C3—H3	0.9800
S1—C3	1.795 (4)	C4—C5	1.504 (5)
O1—C2	1.333 (4)	C4—H4	0.9800
O1—C3	1.425 (4)	C5—H5A	0.9700
O2—C2	1.195 (4)	C5—H5B	0.9700
O3—C6	1.395 (4)	C6—C8	1.483 (5)
O3—C5	1.405 (4)	C6—C7	1.480 (5)
O4—C4	1.408 (4)	C7—H7A	0.9600
O4—C6	1.423 (4)	C7—H7B	0.9600
C1—C2	1.470 (5)	C7—H7C	0.9600
C1—H1A	0.9700	C8—H8A	0.9600
C1—H1B	0.9700	C8—H8B	0.9600
C3—C4	1.490 (5)	C8—H8C	0.9600
			
C1—S1—C3	89.97 (16)	O3—C5—C4	104.7 (3)
C2—O1—C3	113.9 (3)	O3—C5—H5A	110.8
C6—O3—C5	107.4 (3)	C4—C5—H5A	110.8
C4—O4—C6	109.3 (2)	O3—C5—H5B	110.8
C2—C1—S1	107.7 (3)	C4—C5—H5B	110.8
C2—C1—H1A	110.2	H5A—C5—H5B	108.9
S1—C1—H1A	110.2	O3—C6—O4	103.9 (3)
C2—C1—H1B	110.2	O3—C6—C8	111.5 (3)
S1—C1—H1B	110.2	O4—C6—C8	109.2 (3)
H1A—C1—H1B	108.5	O3—C6—C7	109.1 (3)
O2—C2—O1	119.9 (3)	O4—C6—C7	108.8 (3)
O2—C2—C1	126.4 (3)	C8—C6—C7	113.8 (4)
O1—C2—C1	113.7 (3)	C6—C7—H7A	109.5
O1—C3—C4	109.0 (3)	C6—C7—H7B	109.5
O1—C3—S1	106.8 (2)	H7A—C7—H7B	109.5
C4—C3—S1	113.7 (3)	C6—C7—H7C	109.5
O1—C3—H3	109.1	H7A—C7—H7C	109.5
C4—C3—H3	109.1	H7B—C7—H7C	109.5
S1—C3—H3	109.1	C6—C8—H8A	109.5
O4—C4—C3	108.7 (2)	C6—C8—H8B	109.5
O4—C4—C5	104.0 (3)	H8A—C8—H8B	109.5
C3—C4—C5	114.5 (3)	C6—C8—H8C	109.5
O4—C4—H4	109.8	H8A—C8—H8C	109.5
C3—C4—H4	109.8	H8B—C8—H8C	109.5
C5—C4—H4	109.8		
			
C3—S1—C1—C2	19.2 (3)	S1—C3—C4—O4	−57.3 (3)
C3—O1—C2—O2	168.2 (3)	O1—C3—C4—C5	−54.1 (3)
C3—O1—C2—C1	−13.0 (4)	S1—C3—C4—C5	−173.1 (2)
S1—C1—C2—O2	171.1 (3)	C6—O3—C5—C4	28.4 (3)
S1—C1—C2—O1	−7.6 (4)	O4—C4—C5—O3	−11.8 (4)
C2—O1—C3—C4	−96.1 (3)	C3—C4—C5—O3	106.7 (3)
C2—O1—C3—S1	27.2 (3)	C5—O3—C6—O4	−33.7 (3)
C1—S1—C3—O1	−26.0 (3)	C5—O3—C6—C8	83.8 (3)
C1—S1—C3—C4	94.2 (3)	C5—O3—C6—C7	−149.6 (3)
C6—O4—C4—C3	−130.9 (3)	C4—O4—C6—O3	25.9 (3)
C6—O4—C4—C5	−8.5 (3)	C4—O4—C6—C8	−93.2 (4)
O1—C3—C4—O4	61.7 (3)	C4—O4—C6—C7	142.0 (3)

### Hydrogen-bond geometry (Å, °)

**Table:**

*D*—H···*A*	*D*—H	H···*A*	*D*···*A*	*D*—H···*A*
C1—H1A···O3^i^	0.97	2.58	3.428 (4)	146
C1—H1B···O2^ii^	0.97	2.41	3.306 (6)	153
C3—H3···O2^iii^	0.98	2.55	3.265 (4)	129

Symmetry codes: (i) *x*, *y*, *z*−1; (ii) −*x*+2, *y*+1/2, −*z*+1; (iii) *x*−1, *y*, *z*.
